# Adverse effects of the aesthetic use of botulinum toxin and dermal fillers on the face: a narrative review^☆^

**DOI:** 10.1016/j.abd.2024.04.007

**Published:** 2024-11-29

**Authors:** Érico Pampado Di Santis, Sergio Henrique Hirata, Giulia Martins Di Santis, Samira Yarak

**Affiliations:** a Postgraduate Program in Evidence-Based Health, Escola Paulista de Medicina, Universidade Federal de São Paulo, São Paulo, SP, Brazil; b Department of Medicine, Faculdade de Medicina, Universidade José do Rosário Vellano/Universidade de Alfenas, Alfenas, MG, Brazil

**Keywords:** Biocompatible materials, Botulinum toxins, type A, Dermal fillers, Disease notification, Drug-related side effects and adverse reactions, Health planning guidelines, Hyaluronic acid

## Abstract

**Objective:**

To evaluate the adverse effects of facial aesthetic treatments using botulinum toxin and biomaterial implants.

**Methods:**

The bibliographic research for this narrative review considered articles published in journals from the Medline, Pubmed, Embase and Lilacs databases with the following terms: “dermal fillers AND complications, vascular complications AND dermal fillers, adverse reaction, AND toxin botulinum and adverse reaction AND dermal fillers”. Inclusion criteria were articles available in English on adverse events with the aesthetic use of botulinum toxin and dermal fillers/biostimulators.

**Results:**

The demonstration of complications increases simultaneously with the progressive performance of facial aesthetic procedures. Quantitative statistics of the procedures and the countries that use them are skillfully classified, as well as the prosperity trends of these procedures. Complications do not receive the same relevance. There is a deficiency in dissemination of the information by the scientific community, or in other words, there is a publication bias in favor of successful results as opposed to adverse events.*Conclusion*: The lack of knowledge about complications arising from so widely publicized and performed procedures prevents the development of evidence-based guidelines. Complications in aesthetic procedures have become a public health problem, an epidemic that occurs under the supervision of health authorities. Mandatory reporting of adverse events occurring in aesthetic procedures that require medical care aims to fill this gap. With reliable and technical data, it will be possible to identify the causes and perform interventions capable of minimizing irreversible sequelae and deaths. Complications should be promptly recognized by the dermatologist so that, when possible, they can be reversed or adequately managed.

## Introduction

The growing demand for aesthetic procedures, the number of which has increased worldwide,^1^ has made the use of botulinum toxin and biomaterials highly popular.

In 2021, the International Society of Aesthetic Plastic Surgery (ISAPS) released global statistics: 30,439,576 aesthetic procedures, both surgical and non-surgical, were performed.

The application of botulinum toxin continues to lead the non-surgical procedures, with 7,312,616 treatments, which represented an increase of 17.7% compared to 2020.

Biomaterials are natural or synthetic elements, implanted in different parts of the body, especially the face. The aesthetic purpose is to: volumize, repair or even replace tissues. In the case of fillers, the effect is to volumize, while the so-called biostimulators induce inflammatory reactions that culminate in dermal reformulation, especially producing collagen. Hyaluronic acid is recognized for its volumizing effect, calcium hydroxyapatite, and polylactic acid for their fibroblast-stimulating effect.

In 2019, hyaluronic acid used as a filler for aesthetic purposes accounted for 4,315,859 procedures, an increase of 15.7% compared to 2018. And in 2020, hyaluronic acid showed a decrease of 6.1%, probably due to the pandemic, with an absolute number of 4,053,016 applications.

These procedures are characterized by having reduced downtime, and are advertised by professionals, and the authors understanding that as erroneously, as minimally invasive procedures.^2^ The use of terms that attenuate the complexity of medical procedures can hinder the population understanding of the risks they carry.

The industry that manufactures these products aims to commercialize them and acts to pursue their interests. Physicians are not responsible for the marketing, but rather for treatment, performing procedures that improve patients self-esteem and quality of life.^3^ Although these procedures are relatively safe, the increasing occurrence of complications requiring medical intervention is evident.

Physicians should master protocols and guidelines for dealing with adversities so that they can be implemented immediately when complications occur, whether at the time of application or later on. However, there are no scientifically tested guidelines for managing adverse events, despite the clinical trials found in the literature.

Perspectives such as mandatory reporting of adverse events that require medical attention can help to understand the causes of complications and allow the development of evidence-based guidelines.^4^ Thus, future disorders can be prevented and debate can be initiated about products and technologies, techniques, immunological aspects of the host, and the professionals who work at different levels of aesthetic treatments.^2^, ^4^

In this narrative review, the authors address different adverse reactions with recommendations to minimize these reactions when treating patients under use of botulinum toxin, skin implants or biomaterials.

## Methods

This narrative review was based on articles published in journals from the Medline, Pubmed, Embase and Lilacs databases. The following were considered: original articles, experimental studies, case reports, case series, and expert opinions; prospective and retrospective observational (cohort) studies, cross-sectional studies, clinical trials, and literature reviews, with details of individual cases, investigating or discussing the role of biomaterials and botulinum toxin that described adverse events in their use for aesthetic purposes from 2017 to 2022; in addition to publications in textbooks.

The following terms were used: “dermal fillers AND complications, vascular complications AND dermal fillers, adverse reaction AND toxin botulinum and adverse reaction AND dermal fillers, fillers AND autoimmune syndrome”.

The search was limited to full text studies published in English. The screening of eligible articles yielded 50 studies. In addition to these studies, other relevant studies were included in relation to statistical data on adverse reactions to procedures, characteristics of biomaterials and toxins, hypersensitivity reactions, and autoimmune/autoinflammatory syndrome triggered by adjuvants, biofilms, pathogens and medications.

## Adverse events of botulinum toxin, prevention and management

Botulinum toxin (BT) is a protein derived from the bacterium *Clostridium botulinum*. It has high affinity and specificity for neuronal cells, and is capable of binding to these cells, blocking the release of acetylcholine at the motor end plate and causing selective muscle paralysis.^5^

The predisposing factors related to adverse events of the toxin are the quality of the product, characteristics of the diluent, the professional lack of competence (knowledge and skill), and variability of facial anatomy.^6^, ^7^ Adverse events related to BT are less severe when compared to those caused by biomaterials.^8^

## Hematoma and ecchymosis

**Fig. 1:**
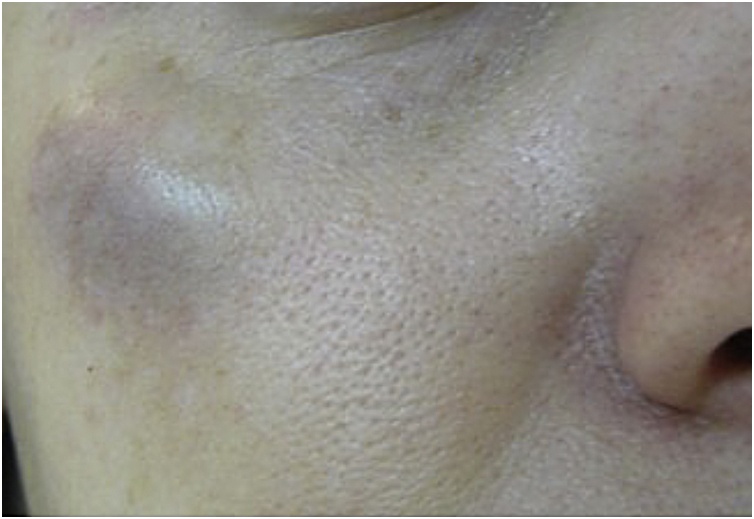
Hematoma in the right orbital arch immediately after application of BT with a 6 × 0.25 mm 31 G needle. There were two previous punctures.

These are the most common adverse effects after the application of BT. Ecchymosis occurs more frequently than hematoma, which is rarer but can occur after applications even with fine needles and a sharp bevel^5^, ^8^ (Fig. 1).

To treat hematomas, analgesics, heparinoid creams, and *Arnica montana* can be administered, although there is no scientific evidence.^8^, ^9^ The patient should be instructed on the decomposition and absorption of the extravasated blood.

## Muscle hypotonia and antagonistic muscle hypertonia

The effect of BT is neurotoxic. When this occurs in the desired muscle targets, a therapeutic effect takes place; however, if the muscle relaxation or paralysis occurs in adjacent muscles or outside the planned area, this effect becomes adverse.^8^ The causes of adverse effects include poor patient selection, injection in an inappropriate location and high doses.^10^ As poor patient selection, one considers patients with unrealistic expectations regarding the true and possible effects of BT, patients with static wrinkles, which do not require muscle movement for clinical presentation and those with high levels, III or IV according to the Glogau aging classification. The clinical signs of aging according to the Glogau classification, described in 1996 at the Seminar on Cutaneous Medicine and Surgery, include, in addition to rhytids, lentigines, keratoses, telangiectasias, shine, vigor and color of the skin of the face. Wrinkles appear at rest in type III, and in type IV wrinkles cover the entire face.^11^

Hypotonia of the levator palpebrae superioris muscle due to poor application or diffusion of the BT leads to ptosis of the upper eyelid.^12^

Brow ptosis depends on the treatment of the frontalis muscle and the obtained adynamia. Eyebrow asymmetry and ptosis are relatively common adverse effects after treatment with BT. Their incidence varies from less than 1% to as high as 5%.^10^

Brow asymmetry occurs when the equivalent dose is not injected on the contralateral side due to inadequate toxin diffusion into the muscle fibers, or even when some fibers of the frontalis muscle are hypertonic in relation to the contralateral ones. This form of asymmetry can be easily controlled by injecting additional units into the hypertonic muscles.^13^

The frontalis muscle has anatomical variations in relation to its lateral planes and the central tendinous plane.^14^, ^15^ A thorough clinical evaluation reduces these unfavorable situations, both due to lack of treatment and overtreatment of the frontalis muscle.^13^

**Fig. 2:**
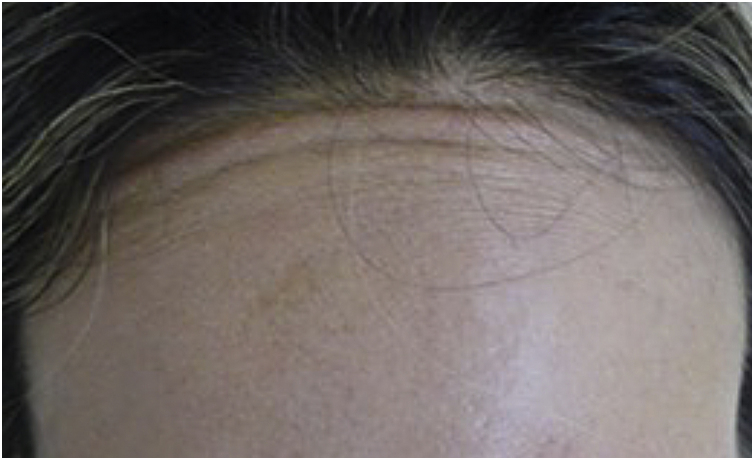
Contraction of occipitofrontalis muscle fibers after treatment of the glabrous portion of the frontal region. Probable lengthening of the superior frontal muscle with relative reduction of the epicranial aponeurosis.

Muscle fibers of the frontalis muscle that go beyond the hair implantation line persist in contraction, causing aesthetic discomfort (Fig. 2).

## Blepharoptosis and ectropion

Blepharoptosis and persistent eyebrow asymmetry may occur after attempts to correct asymmetries. A study of 25 patients submitted to treatment of the frontal region showed an average eyebrow ptosis of 2.3 mm in 22 patients.^15^

Eyebrow ptosis can be prevented by injecting approximately 2 to 3 cm above the supraorbital margin or at least 1.5 to 2 cm above the eyebrow. Eyelid ptosis is observed in elderly people with dermatochalasis who contract the lower fibers of the frontalis muscle to elevate the eyebrows and eyelids.^5^ In patients over 65 years of age treated for glabellar relaxation, asymmetry is an adverse effect that may be observed. Many of these patients have one anatomically lower eyebrow when compared to the contralateral one. This is an asymmetry that is typical of this age group. In summary, the ipsilateral upper eyelid of the lowered eyebrow also has this same characteristic. Compensatory brow elevation will lead to unobstructed vision, but when these patients are treated with BT, the inferior fibers of the frontalis muscle weaken, thus disrupting compensatory brow elevation. Without this elevation, there is an apparent drooping of the upper eyelid, resulting in asymmetry. This can be prevented by carefully examining and considering the baseline asymmetry before determining the dose of BT in the treated area.^15^

Apraclonidine 0.5% eye drops can be used for ptosis as it induces contraction of the Mueller’s muscle, which is a sympathomimetic elevator of the upper eyelid, elevating it 1 to 2 mm.^16^ Phenylephrine ophthalmic solution is an alternative to apraclonidine, but its use should be monitored appropriately due to the risk of adverse effects, such as narrow-angle glaucoma.^5^

**Fig. 3:**
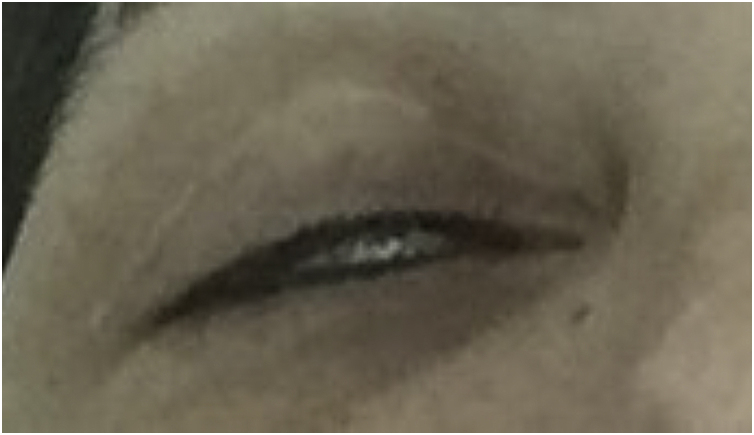
Loss of the ability to close the eyelids due to inadequate relaxation of the fibers of the inferior orbicularis muscle. Note: upper blepharoplasty scar.

When treatment is performed near the lower eyelid, local diffusion of BT may occur leading to a loss of strength in the lateral-inferior orbicularis oculi muscle, which may cause ectropion.^17^ Ectropion can cause complications due to prolonged exposure of the cornea, such as secondary xerophthalmia due to evaporation of the tear film (Fig. 3).

There are no specific treatments for reversing ectropion. The use of ocular lubricants is indicated for cases of xerophthalmia, in addition to ophthalmological monitoring until the physiological reinnervation of the muscle occurs.^13^, ^18^, ^19^

## Headache and ophthalmological changes

Eyelid ptosis is the most common adverse effect, occurring in 3.39% of cases. In the same meta-analysis, headache appears as the second most common adverse effect.^20^

In addition to muscular dystonia, other complications are possible due to the application of BT. The injection of BT into the lateral orbit can cause ophthalmological side effects such as diplopia, ectropion, lagophthalmos, and xerophthalmia.^13^, ^21^

The occurrence of diplopia may be due to the diffusion of BT beyond the orbital septum, which causes relaxation of the extraocular muscles. A study reported an incidence of 1.7% and most of the cases were due to paresis of the inferior oblique muscle.^22^ Corrective lenses with prisms can be used, but the condition is dynamic and temporary, which makes this procedure unnecessary.

Lagophthalmos may occur due to the loss of the sphincter function of the orbicularis oculi muscle, leading to inadequate closure of the eyelids. Loss of sphincter function of the orbicularis oculi muscle, along with eyelid weakness, may occur if the toxin diffuses into the palpebral portion of the orbicularis oculi muscle.

Lubrication in cases of ectropion or lagophthalmos is essential to prevent corneal drying.

Xerophthalmia, due to decreased tear production, may be seen if the toxin is injected deep into the upper lateral periocular area affecting the lacrimal gland.^20^

Epiphora may occur due to toxin-induced weakening of the medial palpebral portion of the orbicularis oculi muscle, with increased tear secretion. These complications can be prevented by the subdermal injection of the toxin and by injecting it laterally to the vertical line that passes through the lateral canthus.^13^, ^18^, ^19^

When planning infraorbital injections, patients with scleral show, those with previous eyelid treatments, or who have xerophthalmia should be excluded, as these may worsen with the application of BT.

Patients with lower septal laxity may show the infraorbital fat pads due to weakening of the orbicularis muscle, making these pseudohernias prominent; therefore, they should not be treated until the pads have been removed.^23^

Reports of dysphagia after the aesthetic use of botulinum toxin in the cervical region are among the adverse effects compiled by the FDA. Extreme cases due to the application of high doses of botulinum toxin present with muscle paralysis of the pharynx and esophagus muscles, causing aspiration pneumonia. Treatment is intubation and assisted breathing.^24^

## Urticaria, anaphylaxis, dyspnea, and soft tissue edema

**Fig. 4:**
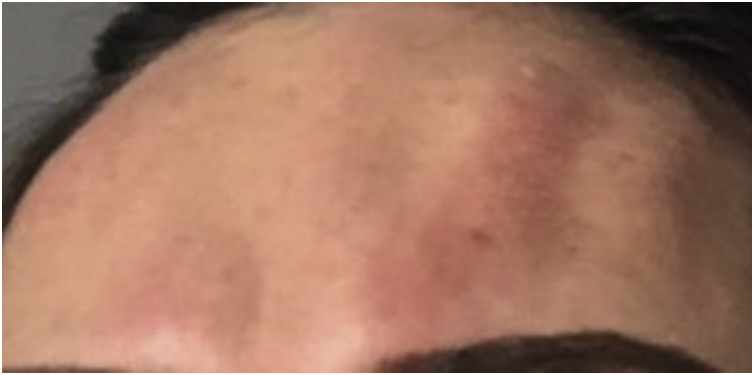
Urticarial reaction immediately after BT application.

Serious complications such as urticaria, anaphylaxis, dyspnea, and soft tissue edema are rare but may occur. Immediate treatment should be initiated (Fig. 4).^25^, ^26^

There have been reports of suppurative granuloma at the site of BT injection that resolved after six weeks of broad-spectrum antibiotic therapy.^26^

Periocular edema lasting a few days may occur. This particular reaction is attributed to lymphatic stasis caused by the weakening of the sphincteric pumping function of the orbicularis oculi muscle, leading to the retention of lymph in the adjacent areas.^26^

Skin infections may occur after BT injection. The injection creates a pore that breaks the protective barrier of the skin and facilitates the penetration of microorganisms. Infections caused by atypical mycobacteria have been reported in the literature.^27^ Atypical mycobacterioses are easily mistaken by common infections and early microbiological testing is indicated for diagnosis. Although rare, this is a serious complication. Lima et al.^28^ reported the emergence of erythematous plaques and nodules with inflammatory signs that appeared days after the application of BT. Five months later, a biopsy was performed and histopathology showed the presence of a granulomatous inflammatory infiltrate in the dermis and hypodermis with epithelioid cells and abscess in the central area. Some acid-fast bacilli were identified by Fite-Faraco staining. Treatment varies in terms of antimicrobials and duration. It may be carried out as monotherapy or require a combination of antimicrobials such as clarithromycin or azithromycin, or a combination of ethambutol and rifampicin^28^ or rifabutin. Minocycline and doxycycline^29^ may be alternatives, as well as sulfamethoxazole associated with trimethoprim. Treatment duration is long and should be maintained for one to two months after clinical resolution.^30^

It is important to closely monitor patients with infectious diseases undergoing cosmetic procedures. Mycobacterial infections are especially challenging because they are difficult to diagnose and treat, and recurrence of the disease is a common morbidity of this process. Treatment of these infections requires medical interventions to achieve cure. Surgical debridement combined with antibiotics according to the antibiogram may be necessary. The three relevant species associated with cosmetic procedures are *Mycobacterium abscessus*, *Mycobacterium chelonae* and *Mycobacterium fortuitum*. *M. abscessus* can form a biofilm and is resistant to treatment.^31^, ^32^

Other adverse events are more common after therapeutic use of BT; however, they can be observed after cosmetic use. The following are highlighted: velopharyngeal insufficiency, brachial plexopathy, generalized muscular hypotonia similar to botulism, necrotizing fasciitis, myasthenia crisis, marked bilateral blepharoptosis, speech and chewing impairment, Guillain-Barré syndrome, dysphagia, aspiration pneumonia, unilateral facial madarosis, alopecia and nephropathy.^33^, ^34^, ^35^, ^36^, ^37^ However, the pathogenesis of neuropathy after botulinum toxin injection is not elucidated, since facial nerve paralysis is a common disease and can occur coincidentally.^31^, ^32^, ^33^ It is known that BT can contribute to the onset of neuropathy because it is capable of binding with high affinity to gangliosides (GT1b and GQ1b), inducing clinical characteristics similar to Guillain-Barré syndrome.^33^, ^34^, ^35^ Neurological examination and electrophysiological evaluation are useful to differentiate facial nerve paralysis from BT-related muscle weakness.^34^

BT and autoimmune antibodies against the thyroid share homology in the amino acid sequence. Epitopes, that is, the smallest portion of the antigen, are found in some homologous regions. These data are relevant because they suggest a possible pathogenetic link between BT and autoimmune thyroid diseases.^38^

It is recommended that patients suspected of having a subclinical neuromuscular disorder be tested before administering pretreatment BT, even at small doses.^39^

Dover et al. report that neurotoxin/BoNT-A protein complexes do not influence immunogenicity. Any relationship between neutralizing antibody formation and clinical response is a complex one, and clinicians must consider other factors that may induce an apparent loss of clinical response.^40^

## Prevention of complications related to botulinum toxin and management of adverse events

Knowledge of facial anatomy, both angiolymphatic and neural and especially muscular, antisepsis techniques; pharmacological principles of BT, interactions with medications and diseases, among other medical knowledge essential for adequate facial treatment with botulinum toxin.^41^, ^42^, ^43^

## Adverse events caused by the use of biomaterials (fillers and/or biostimulators)

Biomaterials are substances derived from or produced by living organisms, or from synthetic materials that interact with the recipients biological system for different purposes, such as supporting and even replacing tissues.^44^ They are widely used for cosmetic purposes.^45^

The FDA (Food and Drug Administration) defines biomaterials used for aesthetic purposes as dermal fillers or injectable implants for the filling of soft tissues, such as the nasolabial fold, malar region, chin, lips and back of the hands. This same agency does not approve their use in the glabella, nose, forehead, neck, breasts and buttocks.^46^ In June 2023, the FDA approved biomaterials for injection in the periorbital region.^47^

The duration of the effect depends on the biomaterial and the area where it is injected. Currently, the most commonly used injectable implants are hyaluronic acid (HA), calcium hydroxyapatite, poly-L-lactic acid,^48^ polymethyl methacrylate and polycaprolactone. They are categorized according to their chemical-physical and rheological characteristics, which include chemical composition, time of permanence, viscosity, cohesiveness and elasticity.^49^

Biomaterials are given the technical name according to the National Health Surveillance Agency (Anvisa, *Agência Nacional de Vigilância Sanitária*), of “intradermal filling solution”, “absorbable implants” and “facial implants”. They are classified as devices and in terms of health risk as: “maximum risk” and, only instructions for use and no leaflets are made available by the Agency. The number of injectable implants for intradermal fillings registered with Anvisa is noteworthy. Hyaluronic acid biomaterials alone account for 121 products. Hyaluronic acid is classified under three technical names, most of which are categorized as “intradermal filling solution” and many of these are recommended by the Regulatory Agency to be injected into the subcutaneous or supraperiosteal region and not intradermally.^50^

Four products based on calcium hydroxyapatite and polycaprolactone are available and registered in the Brazilian market. Three are based on poly-L-lactic acid. Among the polycaprolactone formulations, the technical name is “facial implant”. Products based on calcium hydroxyapatite and formulations with various pharmaceutical principles associated with hyaluronic acid are not registered with Anvisa.

Despite advances in the chemical and biological characteristics of fillers, transient and/or persistent adverse reactions may occur after the use of any biomaterial, even in the hands of experienced dermatologists and plastic surgeons, and may have a substantial impact on the result.^51^, ^52^

All biomaterials are potentially capable of causing serious adverse reactions, although these reactions are not frequent.^53^, ^54^

Some warn that they should only be used by medical doctors others by health professionals. The authors believe that a more homogeneous and clear classification by the competent agencies is necessary and that Law 12.842/2013, in use in Brazil, should be respected.^55^

Most adverse reactions are mild, transient, reversible and non-specific and are related to the procedure and the short-term inflammatory response caused by the implant.^56^, ^57^

However, the scientific community recognizes that adverse reactions are not only associated with technical failure or an increase in the number of indications but also with the immune response that manifests itself in different clinical forms (edema, nodules, indurated plaque) and with different anatomopathological patterns (granulomatous reaction, sarcoid granuloma, foreign body granuloma, eosinophilic panniculitis).^51^, ^58^

The host immune response to the implant may be due to the immunogenicity itself, or to the ability of the biomaterial to act as a superantigen and directly initiate an immune response. The presence of contaminating DNA in injectable implants that, through toll-like receptors (TLRs), can establish the transition from an infected state to a hypersensitive state^59^, ^60^ and induce the production of pro-inflammatory cytokines, such as interleukin-12 and tumor necrosis factor, which are potentially capable of triggering exacerbation of inflammation.^59^, ^61^

Saczonek et al. report that the pathogenesis may be associated with the ability of the biomaterial to behave as an adjuvant because it is capable of binding to standard recognition receptors, triggering an innate immune response. Thus, it is capable of increasing the inflammatory response by interacting with TLRs, activating dendritic cells and inflammasomes with the release of inflammatory cytokines, which can lead to the stimulation of the adaptive immune response and trigger autoinflammatory or autoimmune disease.^62^

Some evidence suggests that prokaryotic organisms (contaminating DNA) can invade the filler implantation area, adhering to the implant surface and allowing the formation of a colony called biofilm. The adaptive behavior of microorganisms within the biofilm and their ability to evade the immune system confers antibiotic resistance.^59^, ^61^

Thus, through several mechanisms, injectable implants are capable of causing undesirable immune-mediated reactions. The risk of these reactions seems to be associated with the length of time the biomaterial remains in the tissues.

### Classification and treatment of adverse reactions to dermal implants

According to Anvisa and WHO, the adverse effects of these devices (biomaterials) are classified as mild, moderate, and severe, according to the percentage of permanence in relation to time, degree of intensity, and frequency in a 30-day period.

Therefore, it is understood that the classification based on the time of onset of signs and symptoms is not adequate and there is no consensus on early and late reactions definitions.

Therefore, the prevalence of complications of injectable implants amenable to treatment is deficient in the scientific literature^63^ and range from low-impact adversities, such as excess product leading to facial asymmetries, to serious, rare and significant adversities that can progress to patients death after infection or vessel occlusion.

It is likely that complications factors may be attributable to the recent and rapid growth in the use of injectable implants, together with inadequate control of the product and the injector. Researchers suggest that to minimize complications, there should be careful consideration of variables such as patient, product and procedure.^64^

Some pre-existing conditions of the patient are absolute or relative contraindications for the use of biomaterials. The following are highlighted: active skin or other infections, for example, sinusitis, periodontal disease, gastroenteritis and urinary tract infection, inflammatory skin conditions (pyoderma gangrenosum) or distant from the treated site (ulcerative colitis, Crohn's disease, osteoarthritis), allergies, uncontrolled diabetes, autoimmune diseases, immunosuppression and coagulation disorders.^61^, ^65^

This article considers the classification used by ANVISA, which is based on the article published by Requena et al.^45^

There are no homogeneous classifications. A mild adverse reaction is considered to be of tolerable intensity and rarely exceeds 30 days. Among the mild adverse reactions to the application of biomaterials, the following are highlighted: ecchymoses /hematoma, erythema, edema, paresthesia, pain and pruritus.

The frequency of ecchymosis/hematoma is variable (1.6 to 51.7%)^66^ and is one of the most common adverse events. Safran argues that it is probably underreported in many lip augmentation articles due to limited patient time. However, it still represents a result that causes discomfort, concern, and prolonged recovery.^63^ There is no specification of the technique used for dermal filling; however, there may be evidence of underreporting when compared with other studies such as that of Daytan, who treated 157 patients and observed hematomas in 93.3%.^67^ Fagien reported the presence of hematoma after lip filling in 92%.^68^ In 2005, Carruthers et al. published a clinical trial where they used hyaluronic acid in the lips of 15 patients. Pain, erythema, and edema occurred in all 15 patients.^69^

On the other hand, Solish et al. filled the lips of 21 patients and 61.5% had edema.^70^ In 2012, Eccleston reported on a group of 60 individuals: 8.3% with edema and nodules at the injection site, while other adverse effects occurred in less than 4% of these 60 individuals. He considered events as mild in 75% and moderate in 25%. Only one case of nodule lasted for more than 12 months.^71^

It is believed that the 100% variation demonstrated by Carruthers and 8.3% by Eccleston may be due to differences in the evaluation method. The same can be considered in the study Beer, who treated 221 patients and obtained severe lip edema in 2%. An additional information in this study is the report of pain in 20%.^72^

In 2013, Fagien observed edema in 94% of patients treated with HA, and these data are in agreement with the findings by Carruthers.^68^, ^69^

Edemas are usually self-limiting.^63^ There is no specific treatment for these adverse reactions, which generally disappear within seven days. Cold compresses, when blood hypoperfusion is completely excluded, are recommended for edema and hematoma, and analgesics/anti-inflammatory drugs for paresthesia. The use of local topical medications for ecchymosis and hematomas lacks scientific evidence.

The Tyndall effect, persistent edema of intermittent or non-intermittent characteristics, non-inflammatory and inflammatory nodules, infection, ulceration, abscess, hypersensitivity reactions, and necrosis are classified as moderate or severe depending on the magnitude of the clinical picture.^73^, ^74^

The Tyndall effect is the appearance of a bluish coloration due to the superficial positioning of the biomaterial, common with hyaluronic acid. Described by John Tyndall in 1868, it is the scattering of light through a dense medium. This dense medium is the filler, with blue light predominating because it is the most distributed wave.^75^ Little is reported about this effect and, if it is not treated when possible, the effect remains visible for several years; hematomas and decreased skin irrigation should be considered in the differential diagnosis.

Persistent, intermittent edemas, classified by some authors as those lasting more than 14 days, cause great aesthetic discomfort. Filling performed on the lower eyelid is prone to this complication. Filling of the tear trough type is susceptible to persistent edema. The authors believe that this area is one of those that requires the most technical knowledge by the physician.

A study conducted by Diwan on the treatment of 48 tear troughs demonstrated that application using blunt-tipped supraperiosteal cannulas without needles proved to be safe. However, the author reports a small number of individuals who developed hematomas and edema that lasted for up to four weeks.^76^

**Fig. 5:**
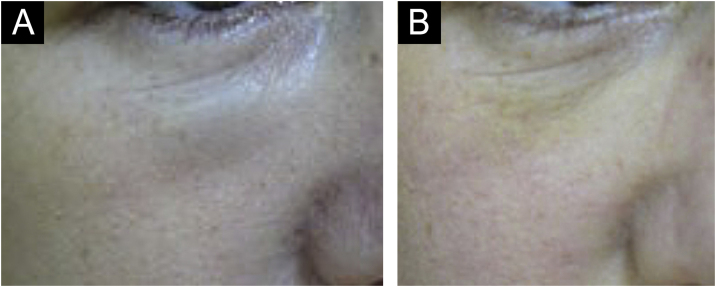
(A and B) Non-inflammatory infraorbital nodule 60 days after hyaluronic acid filling. (B) Four days after hyaluronidase treatment. Note: the brand of the product or the quantity injected were not known.

**Fig. 6:**
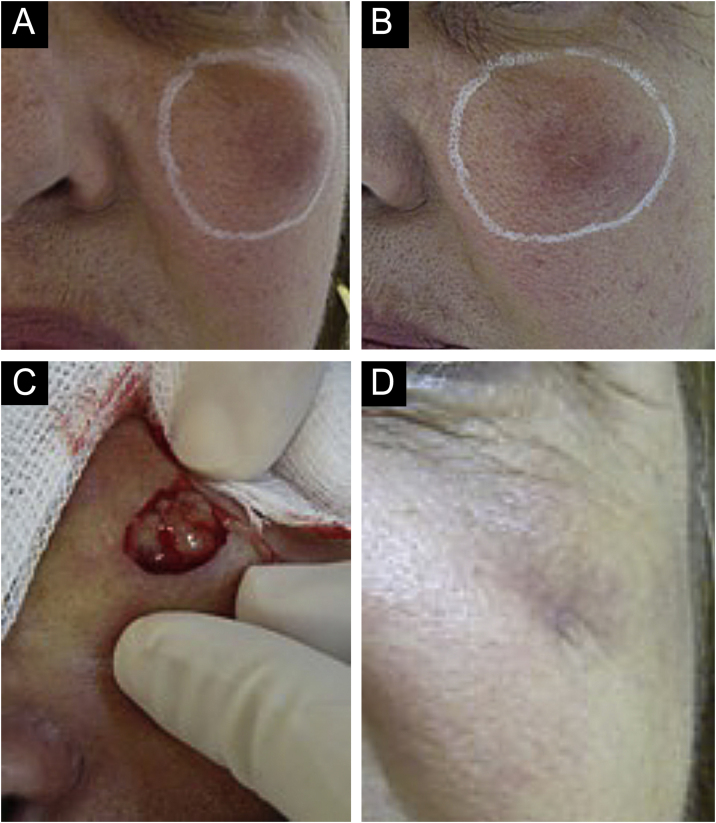
(A‒D) Inflammatory nodule. After drainage with the presence of seropurulent secretion. (A and B) Left malar region. Palpable nodule around the entire perimeter delineated in white. Erythema, edema, increased local temperature and pain on palpation. Onset occurred 20 days after supraperiosteal hyaluronic acid filling, according to information from the professional who performed the procedure. (C and D) Drainage and cleaning of the cavity. Purulent, thick and odorless material. Scar with loss of volume in the drained region. Permanent (severe) adverse effect.

**Fig. 7:**
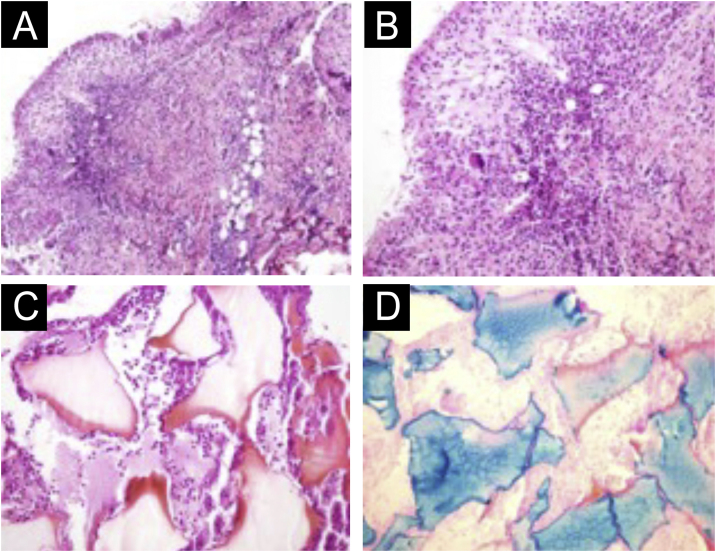
(A‒D) Histopathology of the nodule. (A) Deep reticular dermis with mixed inflammatory and tuberculoid granulomatous infiltrate in palisade surrounding amorphous and eosinophilic material with the characteristics of hyaluronic acid (Hematoxylin & eosin, ×100). (B) Detail showing the palisaded tuberculoid granulomatous infiltrate (Hematoxylin & eosin, ×200). (C) Amorphous and eosinophilic material surrounded by intact and degenerated neutrophil leukocytes (Hematoxylin & eosin, ×200). (D) Strongly positive colloidal iron staining demonstrating hyaluronic acid. Colloidal iron, ×200.

**Fig. 8:**
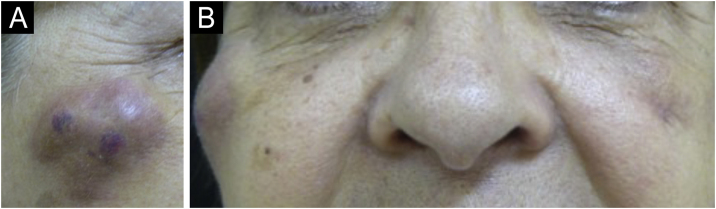
(A‒B) Appearance of nodules in the right malar region, 45 days after the procedure and 30 days after the appearance of the contralateral nodule, with the same characteristics. The patient was already taking broad-spectrum antibiotics due to the presence of the nodule on the left. It is worth noting that there are three nodules, probably at the implant injection sites.

There are statistical variations in relation to the formation of nodules depending on the area and the technique used. Non-inflammatory nodules appear soon after the procedure and represent product accumulation (Fig. 5). Inflammatory nodules represent granulomatous inflammation on histopathology, which may be of infectious or non-infectious origin, with epithelioid cells and giant cells (Langerhans type or foreign body type), lymphocytes, leukocytes, and even plasma cells. Infectious nodules may be caused by fungi, bacteria (Fig. 6) or mycobacteria, and non-infectious nodules present histopathologically as foreign body-type or sarcoidosis-like granulomas (Fig. 7), which may arise years after dermal implantation (Fig. 8).^77^

It is unclear whether late appearing inflammatory reactions (moderate to severe), as named by Artzi, are true hypersensitivity reactions. The experts consulted by Artzi's group strongly supported an infectious etiology or trigger and rejected the word “hypersensitivity” in the context of late inflammatory reactions. They also associated some triggering factors such as viral infections, poor-quality products, combinations of different products, and inadequate techniques. Most of those experts agreed that antibiotics should be administered as the first-line therapy, even more than one antibiotic drug. On the other hand, the use of corticosteroids should be reserved for intense inflammatory reactions with significant edema or for recalcitrant cases and they did not mention the use of antihistamines or anti-inflammatory drugs.^73^, ^78^

**Fig. 9:**
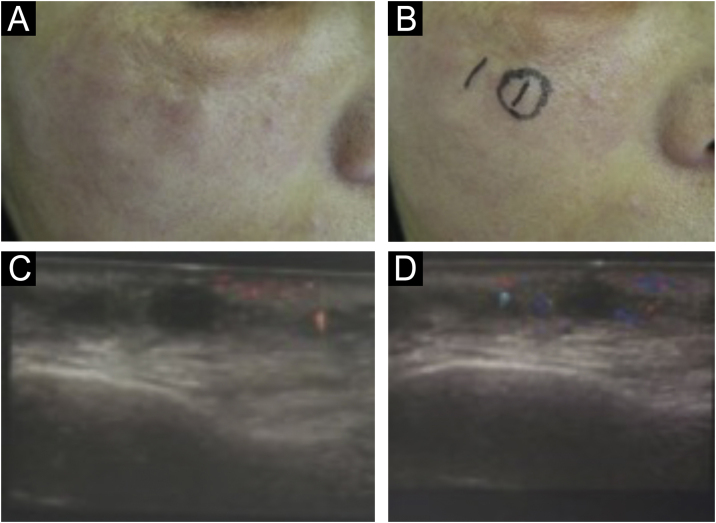
(A‒D) Inflammatory reactivation in sites treated with biomaterial after distant infection, in this case, tonsillitis. (C and D) Ultrasound appearance: increased vascularization around the biomaterial.

It is possible that infections in other regions and traumas may act as triggering factors by allowing small bacteremias (Fig. 9). It has been observed that some nodules develop into abscesses with fistulas and negative cultures. Antibiotic therapy failure suggests the presence of mycobacteria or biofilms of common bacteria, for example, staphylococci. It is not clear how to identify biofilms in these implants and long-term prospective and randomized studies with strict inclusion and exclusion factors are necessary.

In a case report that analyzed lip fillers, the formation of nodules was the most common adverse event, representing 56.7% of cases. The author reports that its incidence is quite rare and is often caused by a greater volume of product placed superficially or in the same spot or by the individual defense reaction, when foreign body granulomas are found on histopathology.^67^

There are no scientific testes guidelines to treat nodule formation, but there are satisfactory results with established medical treatments.

Initially, it is necessary to clinically classify the nodules as inflammatory or non-inflammatory. The inflammatory nodules should then be classified according to clinical characteristics as infectious or non-infectious. Whenever possible, with knowledge of the injected product.

Treatment should be initiated early for non-inflammatory nodules characterized by excess injected material. When the biomaterial is hyaluronic acid, local massage and injection of hyaluronidase (preferably guided by dermatological ultrasound) are recommended. In cases of nodules caused by calcium hydroxyapatite, Reddy mentions laser therapy, massage and injection of saline solution into the nodule; however, good results are not always achieved. Massage and infiltration with saline solution are also mentioned for cases in which the biomaterial used was poly-L-lactic acid.^79^, ^80^

Farwick et al. state that low molecular weight hyaluronic acid has greater inflammatory activity.^81^ However, hyaluronic acid derived from *Streptococcus equi* has a high molecular weight due to the polymer size, which may explain why different fillers, based on HA with different crosslinking, are associated with different reactions, from moderate to severe.^59^, ^60^

In the presence of an inflammatory nodule with clinical characteristics of infection, a biopsy of the lesion and tests are mandatory: histopathology and culture for aerobic, anaerobic bacteria, and mycobacteria, in addition to the antibiogram. After collecting the material for complementary tests, antibiotic therapy may be implemented and, if necessary, drainage of the abscess may be performed.

There is no standardized treatment for foreign body-type granuloma or sarcoidosis-like lesions, where the infectious agent could not be identified. In the literature, there are reports of corticosteroid infiltration with or without 5-fluorouracil, local application of hyaluronidase (if the biomaterial is hyaluronic acid), and some reports of improvement with angiotensin 1 inhibitors and Jack kinase inhibitors, in addition to surgical excision when possible.^82^, ^83^

**Fig. 10:**
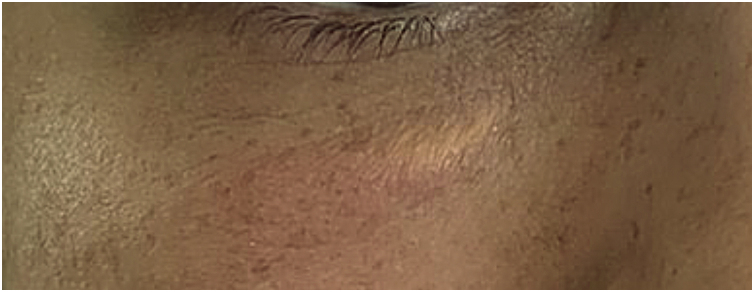
Xanthelasma-like lesion.

Adverse reactions with lesions similar to xanthelasma (Fig. 10) caused by biomaterials is not fully understood. It is believed that the biomaterial can bind to low-density lipoproteins and the LDL-glycosaminoglycan complex is internalized by macrophages. Treatment consists of surgical excision.^84^, ^85^

Anaphylactic shock, migration of biomaterial, permanent nodules, blood supply obstruction due to occlusion in intravascular injections resulting in necrosis, blindness, and stroke are classified as serious and potentially irreversible complications.^73^, ^74^

**Fig. 11:**
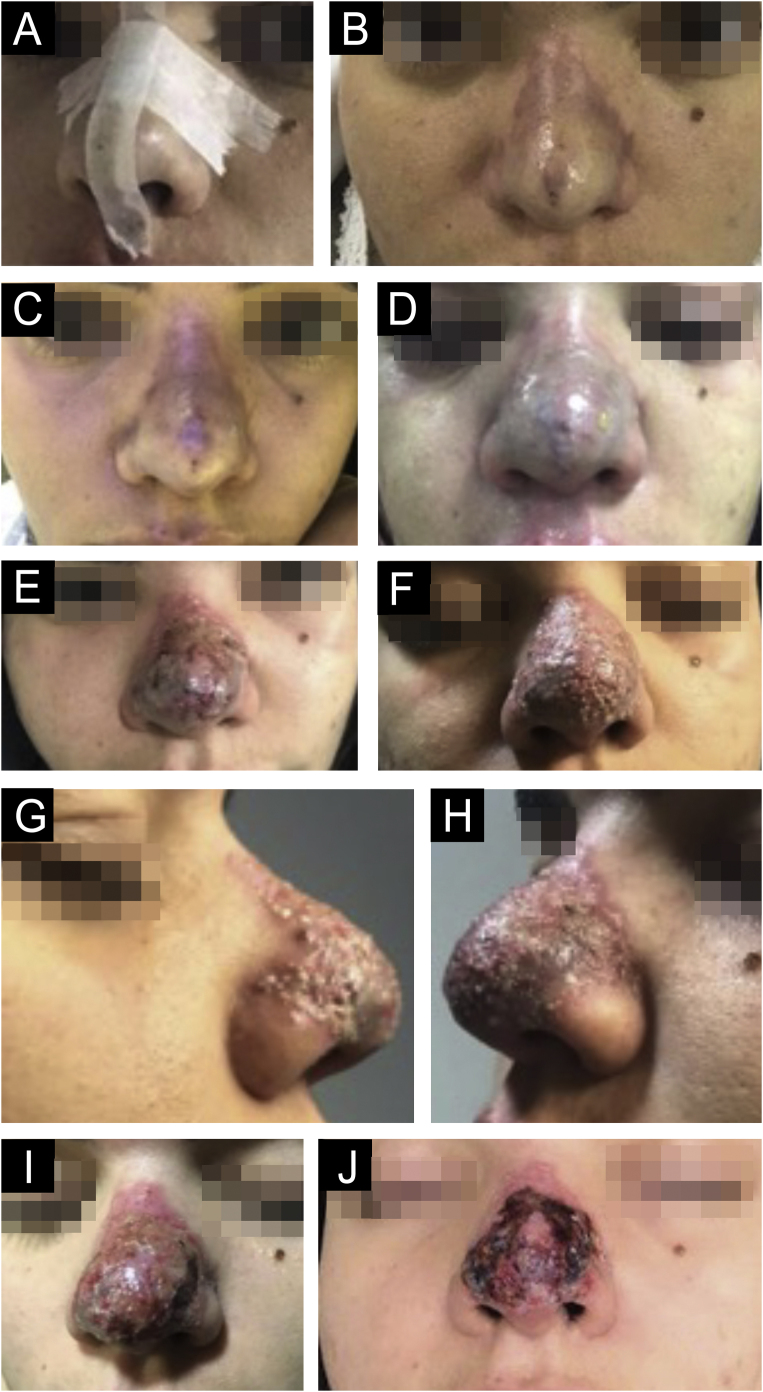
(A‒J) Evolution since the day of application (A); tissue suffering (B‒D). (E–J) Inflammation and necrosis.

Vascular occlusion is one of the most feared complications with biomaterial fillers. Authors have confirmed that both needles and cannulas can lead to vascular complications (Fig. 11). However, there seems to be a decrease in the risk of stroke with blunt-tipped cannulas and smaller gauges; however, one should not overestimate the safety of filling using a cannula.^86^, ^87^

Attention must be paid to minor signs of vascular occlusion. They may be misdiagnosed as hematoma, pain, and edema related to the injection and have their treatment delayed.^88^

Doerfler et al. found that 25 patients among 28 cases of severe embolism due to hyaluronic acid used 27 G to 22 G cannulas. Among these patients, nine cases evolved to blindness, one case to blindness with stroke, and 18 cases to large-area necrosis. The authors concluded that cannulas with a gauge smaller than 25 G have a lower risk of stroke.^89^, ^90^

The countries that reported the most cases of vascular occlusion were Korea and China.^91^ These data may suggest a lack of knowledge about the actual complications. In 2019, 398,830 HA applications were performed in Brazil; 303,812 in Italy; 192,358 in France; 170,515 in Mexico, and 140,795 in Turkey.^92^ There were no published cases of these complications in the aforementioned countries, which strongly suggests underreporting.

Lee states that although slow injections with low pressure are generally considered safe, the differences in ejection pressure during a filler injection still need to be determined. He concludes that regardless of the injection force, the ejection pressure is probably higher than the vascular pressure at the time of entry into the vessel, which makes the injection dangerous. Even the lowest ejection pressure was higher than the blood pressure. It was observed that the lowest pressure calculated at the time of the injection was at least five times higher than the systolic blood pressure.^93^

The underreporting and non-publication of vision loss complications challenge the credibility of the known data, making it difficult to plan for the prevention of complications.^4^

The ratio between the complications reported in six years in relation to the number of applications in just one year, 2019, is not credible. Brazil, with 398,830 applications, did not report any vision loss publications; Thailand, with 4,733, reported six cases of this complication.

**Fig. 12:**
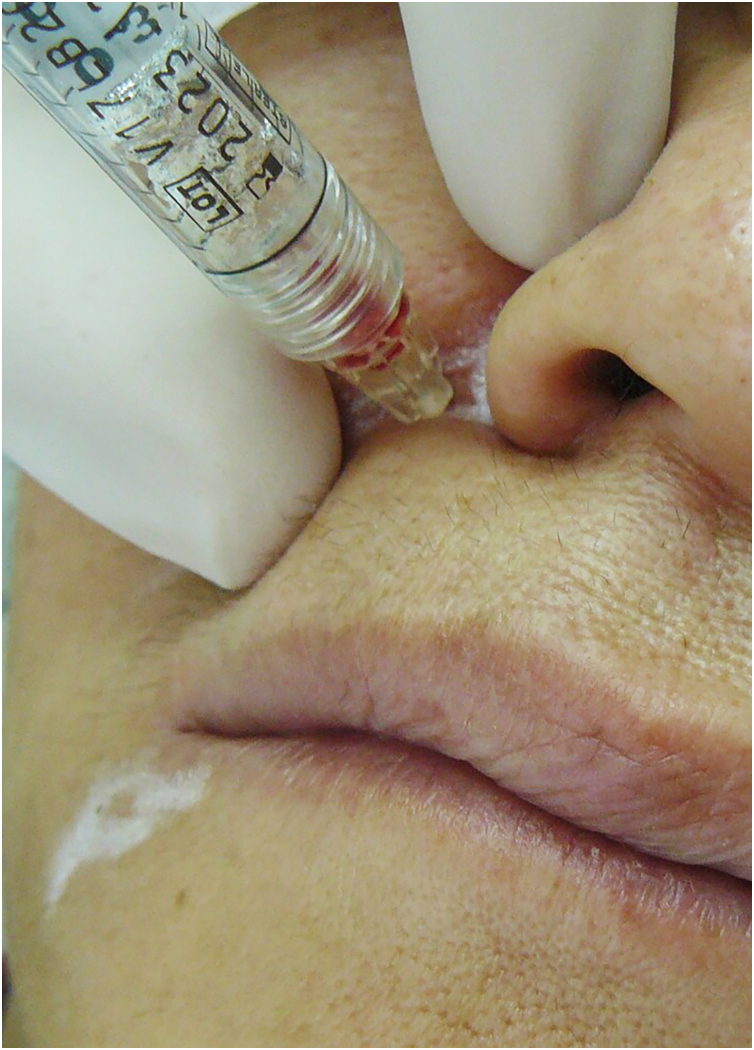
Aspiration positive for blood.

Physicians should minimize the risks of vascular complications by mastering the vascular anatomy of the face.^89^ A careful technique, using low injection pressure and prior and prolonged aspiration should be performed, but it does not exempt from this fateful adversity. Regarding aspiration before injection, there seems to be low reliability in negative aspirations (Fig. 12).

Evidence published by Casabona^94^ and Van Loghem^95^ showed, respectively, that the reliability of aspiration is 53% according to Casabona, and between 33% and 63% according to Van Loghem.

This fact shows that it is not possible to establish how to perform an ideal and safe aspiration before injection. However, these authors recommend systematic aspiration with the needle at the injection site with negative pressure for ten seconds, slow injection with dispensing of a small volume of filler, and attention regarding discomfort/pain, signs of skin discoloration overlying the treated area, and intervals between one region and another. The need for constant dialogue about pain and discomfort and the paradox that truncal anesthesia or sedation can inhibit this body defense reflex make the procedure riskier.

The early diagnosis of vascular occlusion and treatment within 48 hours yields the best results.^96^

The need for legislation to make the reporting of cases with complications that require the intervention of a medical professional mandatory is emphasized.^2^, ^4^

Cerebrovascular accident (CVA) or stroke is an even rarer complication resulting from intra-arterial injection of fillers. Moore et al. demonstrated the case of a patient who suffered three complications after the injection of hyaluronic acid: vascular involvement of the frontal skin, unilateral blindness, and ipsilateral subclinical strokes. If it were not for a stroke investigation protocol, these complications might not have been diagnosed. It is suggested that the incidence of stroke due to intra-arterial injection may be higher than that reported.^97^

The use of dermal fillers, mainly hyaluronic acid, in the periocular area is increasing – both for functional and aesthetic indications, as they offer an alternative to some surgical procedures with the advantage of instant results, minimal recovery time, and low complication rates. However, success depends on the careful selection of patients, products, and procedures to achieve favorable results. All implants can induce tissue ischemia; although small cutaneous vascular occlusions are not uncommon, cases of blindness secondary to facial filler injections are considered rare.^98^

Signs and symptoms of tissue necrosis may occur during or hours after the injection with a clinical picture similar to Nicolau syndrome (immediate pain, pallor and livedo and after a few days erythema, edema, pustules with neovascularization, and skin necrosis).^99^, ^100^

Several theories have been proposed for the pathogenesis of tissue necrosis induced by the injection of dermal implants. The mechanism of inflammation and cell destruction is not well understood and it seems that the final lesion is due to the combination of three factors: thrombosis and embolism, angiospasm due to edema and local inflammatory reaction and vascular endothelium injury and/or arterio-arterial or veno-arterial reflexes. Other authors add a vasculitis-type immune-allergic response with deposition of immune complexes, complement activation and neutrophil chemotaxis, resulting in necrosis.^101^, ^102^

An animal model showed that it is possible to produce tissue necrosis when, in addition to arterial embolism, there is obstruction of the flow of terminal arteries that irrigate the region through anastomoses.^103^

Regarding the conduct to be taken in the case of cutaneous vascular ischemia, there are only circumstantial treatments and conducts with a low level of evidence.

The level of evidence supporting the use of hyperbaric oxygen therapy in acute complications of fillers is generally weak.^86^ The use of high-dose hyaluronidase in the first 24 hours shows better results in skin ischemia than after 24 hours, despite the lack of evidence.^96^

On the other hand, there is no evidence of its use in preventing blindness and treating stroke.^98^

There is no direct evidence that acetylsalicylic acid (ASA) prevents platelet aggregation in the case of an occlusion related to hyaluronic acid. Some authors consider the use of ASA to be reasonable and safe, based on the extrapolation of evidence from acute coronary syndrome.^104^ There is no evidence that nitroglycerin paste helps with HA obstructions.^105^ In filler-induced tissue ischemia, the filler product is present in the arterioles. Theoretically, early application of nitroglycerin paste might not improve perfusion and worsen ischemia with dilation of vessels and subsequent spread of the product to smaller arterioles and capillaries.^106^

There are no comparative studies measuring the results and efficacy of peripheral vasodilators on facial microcirculation. Sildenafil has been used in non-erectile disorders. As it is a selective inhibitor of c-GMP-specific phosphodiesterase type 5, it promotes relaxation of the smooth muscle of the corpora cavernosa and dilation of the arteries, providing c-GMP-dependent macrovascular and microvascular dilation. However, as capillaries do not have smooth muscle cells, capillary flow speed depends mainly on the vasomotor tone in the arterioles and their relaxation results in better capillary filling and increased flow. On the other hand, the short half-life of sildenafil (approximately four hours) limits its clinical use. Some authors have shown that sildenafil contributed to improving microcirculation and symptoms of Raynaud's phenomenon and the viability of skin flaps through its vascular effect and inhibition of platelet activity. However, one cannot assess whether its use would be beneficial in cases of vascular injury caused by dermal fillers.^107^, ^108^

Low molecular weight heparin (LMWH) is the most indicated due to its anticoagulant and anti-inflammatory properties, mainly helping to relieve pain.^109^ Due to its anti-inflammatory action, it reduces vasospasm and, thus, improves local arterial flow, in addition to preventing the formation of microthrombi caused by vascular injury, due to its anticoagulant action. For better anti-inflammatory action, corticosteroids are associated with an anti-inflammatory dose for three to five days; however, there is no scientific evidence.

Therefore, since there is no established standard for the treatment of vascular occlusion, the most important thing is prevention, technical quality in the procedure performance, and its follow-up after early, outpatient, and late intervention. The patient must have contact with the performing physician or a member of the team because since is a specific procedure, not all emergency services will have the technical conditions to implement early treatment, which is essential for a successful prognosis.

## Autoimmune/inflammatory syndrome induced by adjuvants (ASIA syndrome)

ASIA syndrome was described in 2011, first by Shoenfeld et al.^110^ and involves a single group of immune-mediated diseases that can probably develop in genetically predisposed individuals after exposure to adjuvants (stimuli or triggering factors that trigger immune activity), with a latency period and clinical spectrum of siliconosis, Gulf War syndrome, macrophagic myofasciitis syndrome and post-vaccination phenomena.^110^ These conditions share several clinical features with the possible appearance of autoantibodies and a tendency to improve when the triggering factor is removed. Diagnostic criteria suggested by the authors, listed in Table 1, for the diagnosis of the ASIA syndrome correspond to two major criteria, or to one major criterion and two minor criteria.^111^

**Table 1:** Diagnostic criteria for ASIA syndrome.^111^

Major	Minor
Exposure to external stimulus	Autoantibodies against adjuvants
Latency: variable	Other clinical manifestations
Clinical manifestations	HLA specific (DRB1, DQB1)
Local: nodules/edema	
Systemic: nonspecific (e.g., myalgia, myositis, muscle weakness, arthralgia, foreign body granuloma in lymph nodes, arthritis, chronic fatigue, sleep disturbance, neurological manifestations, cognitive impairment, memory loss, fever, dry mouth)	
Removal of the inducing agent induces improvement	Autoimmune disease arises
Typical histopathology of the involved organ	

Alijotas et al.^59^ and Cohen et al.^112^ associated new adjuvants to ASIA syndrome. These include injections of biomaterials other than silicone, such as hyaluronic acid, acrylamides, methacrylate, and other injectable oils. In 2013, Alijotas et al.,^59^ suggested the inclusion of histocompatibility antigens (HLA B8, DRB1, DR3, DQB1 E) to the major criteria of the syndrome and expanded the minor criteria (increased gamma globulins and/or LDH and/or angiotensin-converting enzyme and decreased complements) with laboratory tests.^59^ And more recently, in 2023, Cohen et al.^112^ added dysregulated non-classical autoantibodies directed against G protein-coupled receptors (GPCRs) of the autonomic nervous system and small fiber neuropathy (SFN), in an attempt to explain the development of dysautonomia in ASIA syndrome.

In the review carried out by Borba et al., in 2020,^113^ the authors emphasize that the etiology of the clinical conditions of ASIA Syndrome is far beyond understanding, relating sarcoidosis, Sjögren's syndrome, undifferentiated connective tissue disease (UCTD), silicone implant incompatibility syndrome, and adverse events related to the immune system as classic examples of ASIA Syndrome. Thus, these authors believe that it is possible to assume autoimmunity/autoinflammation in these diseases due to major (clinical) and minor (immunogenetic) criteria. Other authors^112^, ^113^ suggest that patients who show adverse reactions to biomaterials should be monitored for several months.

The authors believe that the presence of triggering factors with immunological activity (or adjuvants, such as infectious agents, dust, vaccines, silicone, aluminum salts, biomaterials, among others) and cooperation in the favorable and genetically determined context are necessary to promote disease onset. However, there is no time limit between exposure to these adjuvants and the effect (ASIA syndrome) and it is difficult to prove a causal relationship between complaints and exposure that occurred several years earlier.^114^ On the other hand, the authors consider it important to alert dermatologists to the possible occurrence of the syndrome, albeit rare, since these professionals are qualified to treat these adverse reactions.

### Strategies for preventing complications

Pre-, during and post-treatment recommendations are considered important to minimize adverse events and inform patients about the risks of injectable implants. The following considerations is noteworthy: mandatory medical consultation prior to the procedure. The prior consultation consists of a physical examination, anamnesis, previous history of the personal and family illnesses, pregnancy, use of medications, supplements,vitamins and previous procedures. Besides a fair explanation of the procedure to be performed, its benefits and risks should be provided. It is part of the medical doctor ethical and professional conduct to inform the patient of the diagnosis, prognosis, risks and objectives of the treatment. All medical doctors are encouraged to present this information in writing, explain it to the patient, and have the informed consent form signed by the patient, including the possibility of treatment for possible complications.

Dermatological ultrasound can complement the clinical examination to identify possible previously implanted biomaterials, in addition to showing the vascular structures and treatment planes. It is also important to guide the treatment of adverse reactions, by revealing abscesses, and suggesting the most appropriate sites for hyaluronidase infection and biopsy.

As for the product, it is important to be aware of its license with the appropriate agencies and to be aware of possible interactions with other biomaterials.

As for the application, after photographic documentation, the application planning should be carried out with the quantity and description of the product, knowledge of anatomy, appropriate technique for the anatomical region, and training. The procedure must follow the aseptic surgical technique recommended in medical schools.

Given the scarcity of long-term prospective randomized clinical trials (trials generally last one year) and in light of the underreporting of complications, there is a lack of information in the literature on the efficacy and safety of these procedures.^63^

The development of guidelines with data from the compulsory notification of complications arising from aesthetic procedures that require medical assistance will provide real knowledge of the causes and may provide the basis for future research, classification of severity, and also developing knowledge about the prognosis of these complications. Bill number 9.602/2018, approved by the Health, Social Security, and Family Committee on its merits and by the Constitution and Justice and Citizenship Committee on its constitutionality and legality, provides for this notification throughout the Brazilian territory.^4^ This Bill is currently under consideration for approval by the Federal Senate of Brazil.

## Conclusion

The authors disagree with the term “minimally invasive” used by professionals and even in articles published in scientific journals. Classifying procedures that are certainly invasive, such as botulinum toxin and the use of biomaterials for aesthetic purposes, as “minimally invasive” can lead to confusion and give the layperson the false impression that there are no adverse effects, which is not true.

Medical doctors should be familiar with protocols and guidelines for dealing with adversities so that they can be implemented immediately when complications occur at the time of application.

Perspectives such as mandatory reporting of adverse events caused by aesthetic procedures that require medical care can help to understand the causes of complications and allow the development of evidence-based guidelines,^4^ so that future complications can be effectively prevented.

It is essential to have systematic and organized knowledge of the causes that give rise to complications. The authors understand that notification and recognition of causes will help in the prevention and treatment and will encourage debate about products, technologies, techniques, and qualification of professionals performing aesthetic procedures.

## Authors' contributions

Érico Pampado Di Santis: Design and planning of the study; collection of data, or analysis and interpretation of data; drafting and editing of the manuscript or critical review of important intellectual content; effective participation in research orientation; critical review of the literature; approval of the final version of the manuscript.

Sérgio Henrique Hirata: Drafting and editing of the manuscript or critical review of important intellectual content; effective participation in research orientation; critical review of the literature; approval of the final version of the manuscript.

Giulia Martins Di Santis: Collection of data, or analysis and interpretation of data; critical review of the literature.

Samira Yarak: Design and planning of the study; collection of data, or analysis and interpretation of data; drafting and editing of the manuscript or critical review of important intellectual content; effective participation in research orientation; critical review of the literature; approval of the final version of the manuscript.

## Financial support

None declared.

## Conflicts of interest

None declared.
